# Up-Regulation of S100A11 in Lung Adenocarcinoma – Its Potential Relationship with Cancer Progression

**DOI:** 10.1371/journal.pone.0142642

**Published:** 2015-11-06

**Authors:** Tetsukan Woo, Koji Okudela, Hideaki Mitsui, Michihiko Tajiri, Yasushi Rino, Kenichi Ohashi, Munetaka Masuda

**Affiliations:** 1 Department of Surgery, Yokohama City University Graduate School of Medicine, Yokohama, Kanagawa, Japan; 2 Department of Thoracic Surgery, Saiseikai Yokohama City South Hospital, Yokohama, Kanagawa, Japan; 3 Department of Pathology, Yokohama City University Graduate School of Medicine, Yokohama, Kanagawa, Japan; 4 Department of Thoracic Surgery, Kanagawa Cardiovascular and Respiratory Center, Yokohama, Kanagawa, Japan; University of North Carolina School of Medicine, UNITED STATES

## Abstract

We previously reported that patients with lung adenocarcinomas with KRAS gene mutations and strong proliferating activity had poorer outcomes, even in the early stage of the disease. The aim of the present study was to elucidate the potential molecular basis of these highly malignant lung tumors by focusing on S100 proteins (S100A2, S100A7, and S100A11), which are downstream targets of oncogenic KRAS and promoters of tumor progression. The immunohistochemical expression of S100 proteins was examined in 179 primary lung adenocarcinomas, and the potential relationships between their levels and clinicopathologic factors were analyzed. Among the three subtypes, S100A11 levels were significantly higher in adenocarcinomas with KRAS mutations and strong proliferating activity. They were also higher in adenocarcinomas with poorly differentiated tumors. Furthermore, higher levels of S100A11 were associated with shorter disease-free survival. These results suggest that the up-regulation of S100A11 plays a role in tumor progression, particularly in KRAS-mutated lung adenocarcinomas.

## Introduction

Lung cancer is one of the most common causes of cancer-related death in the developed world [1–2]. A large proportion of patients, even those with early stage non-small-cell lung cancer, die due to recurrent disease [3–4]. Although some lung tumors are sensitive to conventional chemotherapeutic agents or certain molecular targeting agents, many are not [5–6]. Thus, a deeper understanding of the molecular basis of lung carcinogenesis is needed in order to develop novel therapeutic strategies.

We previously reported that patients with lung adenocarcinomas with KRAS gene mutations and strong proliferating activity had poorer outcomes, even in the early stage of the disease [6]. Therefore, the aim of the present study was to elucidate the potential molecular basis of these highly malignant lung tumors by focusing on S100 proteins. The S100 protein family binds to calcium and modulates the transmission of various cellular signals. We previously demonstrated that S100A2, S100A7, and S100A11 were downstream targets of oncogenic KRAS (Tables 1 and 2) [7–8]. The expression of S100 proteins was also shown to be altered in various cancers [9–20] (laryngeal [10], breast [11], lung [12–13], gastric [14], colorectal [15], hepatocellular [16], pancreatic [17], prostate [18], kidney [19], and bladder cancers [20]) including lung cancers [12–13], and these proteins have been suggested to promote the progression of carcinogenesis by modulating cell migration and proliferative activity [21].

**Table 1:** Downstream targets up-regulated by oncogenic KRAS with gene chip microarray analysis.

			**Mock**		**KRAS/G12**		**KRAS/V12**		**Ratio**	
Gene	Accession	Map	Signal Value	Flags	Signal Value	Flags	Signal Value	Flags	V12/G12	V12/MOCK
PPBP	R64130	4q12-q13	0.060	A	0.106	A	24.380	P	230.647	406.330
NAV3	NM_014903	12q14.3	0.180	A	0.307	A	32.910	P	107.024	182.831
IL1RL1	NM_003856	2q12	0.123	A	1.000	A	17.379	P	17.379	141.224
ESM1	NM_007036	5q11.2	0.170	A	0.404	A	22.068	P	54.680	129.812
SERPINB2	NM_002575	18q21.3	0.353	P	0.100	P	36.969	P	36.969	104.676
CCL3	NM_002983	17q11-q21	0.420	A	0.932	A	43.922	P	47.122	104.577
MMP1	NM_002421	11q22.3	0.823	P	1.000	P	82.574	P	82.574	100.284
NICE-1	NM_019060	1q21	0.952	A	1.000	A	82.501	P	82.501	86.700
IL24	NM_006850	1q32	0.205	P	1.000	P	14.916	P	14.916	72.744
IL1B	NM_000576	2q14	0.589	P	1.000	P	32.344	P	32.344	54.881
PI3	NM_002638	20q12-q13	0.270	A	0.327	A	14.277	P	43.698	52.877
S100A7	NM_002963	1q21	0.300	A	0.625	A	9.611	P	15.387	32.035
HSD17B2	NM_002153	16q24.1-q24.2	0.160	A	0.231	A	5.041	P	21.857	31.504
FOS	BC004490	14q24.3	0.480	A	0.951	A	11.526	P	12.116	24.013

Accession, gene bank accession number; Map, chromosome locus; MOCK, mock-transduced NHBE-T; G12, wild-type KRAS-transduced NHEB-T; V12, oncogenic mutant KRAS-transduced NHBE-T.

Flags indicate whether gene expression was present (P) or absent (A). Genes whose signal values were more than 20-fold higher in KRAS/V12 cells than in mock- and/or KRAS/G12 cells are extracted from our date of a gene chip microarray analysis previously described [7]. The genes sorted are listed in this table.

**Table 2:** Downstream targets up-regulated by oncogenic KRAS with proteomic analysis.

				Signal Intensity			Signal Ratio	
Protein	Accession	Map	P value	MOCK	G12	V12	V12/G12	V12/MOCK
S100A2	NM_005978	1q21.33	0.0001214	0.400	1.005	1.665	1.657	4.163
VIME	NM_003380	10p13	0.00000091	0.368	0.754	1.424	1.889	3.870
EEF2	NM_001961	19p13.3	0.00000313	0.454	0.922	1.515	1.643	3.337
GC	NM_000583	4q12-q13	0.001	0.480	0.934	1.582	1.694	3.296
CTSL1	NM_001912	9q21.33	0.0003414	0.635	0.708	1.708	2.412	2.690
VCP	NM_007126	9p13.3	0.002	0.599	1.010	1.551	1.536	2.589
ENOA	NM_001428	1p36.2	0.0001339	0.641	0.936	1.560	1.667	2.434
S100A11	NM_005620	1q21	0.002	0.569	0.761	1.291	1.696	2.269
CTSL1	NM_001912	9q21.33	0.001	0.730	0.802	1.551	1.934	2.125

Accession, gene bank accession number; Map, chromosome locus; MOCK, mock-transduced NHBE-T; G12, wild-type KRAS-transduced NHEB-T; V12, oncogenic mutant KRAS-transduced NHBE-T.

Proteins whose signal intensities were more than 2-fold higher in KRAS/V12 cells than in mock- and/or KRAS/G12 cells are extracted from our date of a comprehensive proteomic analysis previously described [8]. The proteins sorted are listed in this table.

We here examined 179 surgically resected primary lung adenocarcinomas for the immunohistochemical expression of S100 proteins (S100A2, S100A7, and S100A11), and analyzed the potential relationships between their levels and clinicopathologic factors.

## Materials and Methods

### Primary Lung Cancer

All 179 lung cancers examined were cases of stage I adenocarcinoma that underwent radical surgical resection at the Kanagawa Cardiovascular and Respiratory Center (Yokohama, Japan). The research plan was approved by the Ethics Committees of Yokohama City University and Kanagawa Cardiovascular and Respiratory Center. Written informed consent for the research use of resected materials was obtained from all subjects providing materials.

Histologic subtypes were classified according to the 2011 IASLC/ATS/ERS classification of lung adenocarcinoma [22], and tumor stages were determined according to the international TNM classification system (seventh edition of UICC)[23].

### Western blotting

Tissue lysates were subjected to SDS-polyacrylamide gel electrophoresis, and transferred onto PVDF membranes (Amersham, Piscataway, NJ). The membranes were then incubated with nonfat dry milk in Tris-buffered saline containing Tween-20 (TBS-T) in order to block non-immunospecific protein binding, and then with a primary antibody against S100 A11 (Santa Cruz, Santa Cruz, CA), and beta-actin (Sigma, St. Louis, MO). After washing with TBS-T, the membranes were incubated with animal-matched HRP-conjugated secondary antibodies (Amersham). Immunoreactivity was visualized with an enhanced chemiluminescence system (Amersham).

### Immunohistochemistry

Tumor sections were cut from formalin-fixed, paraffin-embedded tissue blocks. Sections were deparaffinized, rehydrated, and incubated with 3% hydrogen peroxide, followed by 5% goat serum to block endogenous peroxidase activities and non-immunospecific protein binding. Sections were boiled in citrate buffer (0.01M, pH6.0) for 15 minutes to retrieve masked epitopes and then incubated with primary antibodies against S100A2, S100A7, and S100A11 (Santa Cruz) as well as Ki-67 (DAKO, Ely, UK). Immunoreactivity was visualized using an Envision detection system (DAKO), and nuclei were counterstained with hematoxylin. The immunohistochemical expression levels of S100 proteins were evaluated by a scoring system as described in the Results section. The labeling index of Ki-67 was calculated as the proportion of cells with positive nuclei among 500–1000 cancer cells. Ki-67 labeling indices of <10% and > = 10% were classified as low and high levels based on the findings of our previous study [6].

### Statistical Analysis

Differences in scores among the groups classified based on clinicopathologic subjects were analyzed by the Mann-Whitney or Kruskal-Wallis test. The relationships between scores and clinicopathologic subjects were analyzed using a multiple linear regression model. The post-operative disease-free span was defined as the period ranging from the date of surgery to the date when recurrence was diagnosed. An observation was censored at the last follow-up if the patient was alive or had died of a cause other than lung cancer. Recurrence curves were plotted using the Kaplan-Meier method, and the absolute risk of recurrence at five years was estimated from these curves. Differences in the disease-free survival spans were analyzed using the Log-rank test. P values less than 0.05 were considered significant. All statistical analyses were performed using SPSS software (SPSS for Windows Version 10.0; SPSS; Chicago, IL, USA).

## Results

### Immunohistochemical expression of S100 proteins

**Fig 1:**
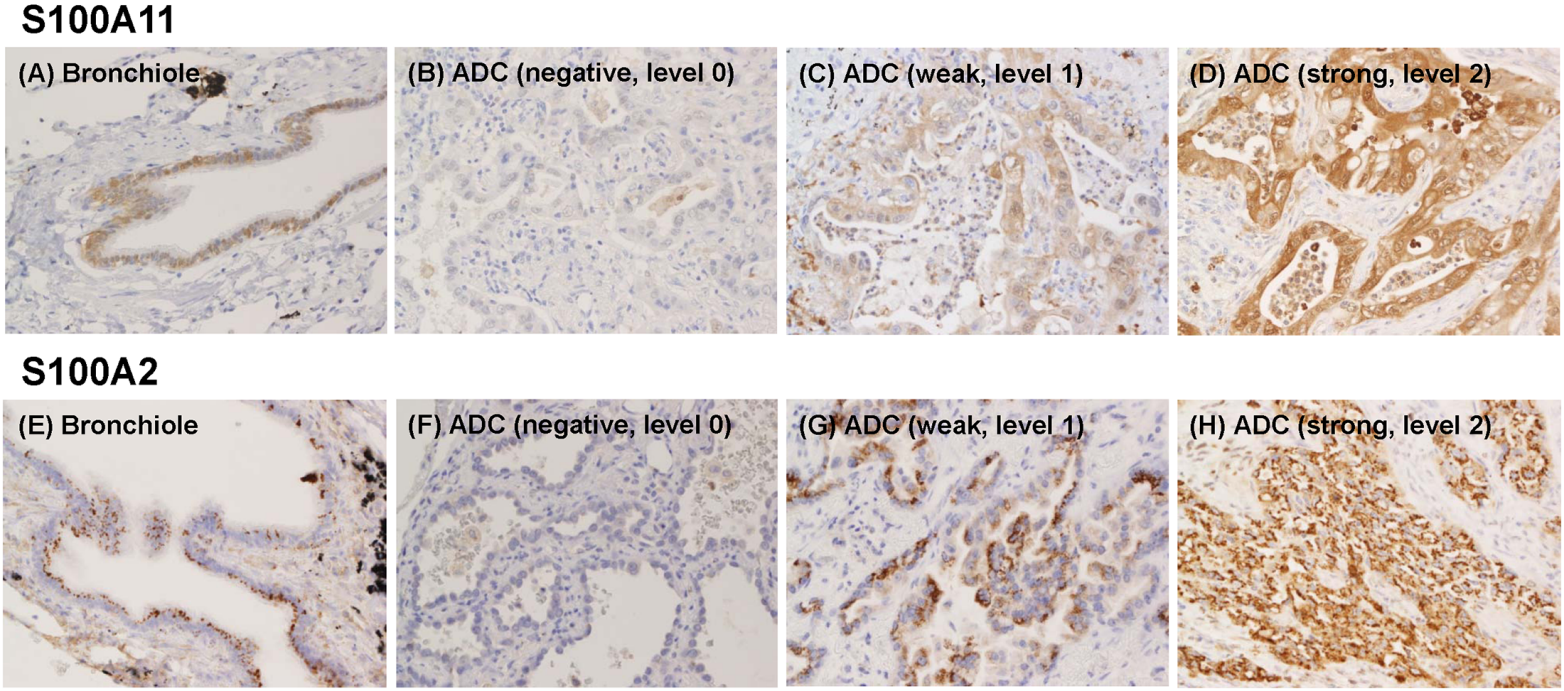
Immunohistochemical examination of S100A11 and S100A2 protein expression levels in tumors and non-tumorous epithelia from lung adenocarcinoma (ADC) patients undergoing surgical resection. Representative photographs from normal bronchioles (A, E) and tumors, in which the expression of S100A11 and S100A2 was negative (B, F), weak (C, G) and strong (D, H), are shown.

The S100A11 protein was expressed in the nuclei and cytoplasm of the normal epithelial cells of bronchioles (Fig 1A), while the S100A2 protein was expressed predominantly on the apical side of the cytoplasm in a granular form (Fig 1E).

S100 protein levels varied in neoplastic cells, and even in individual tumors. The expression of these proteins was higher in some neoplastic cells than in bronchiolar epithelial cells (Fig 1D and 1H), whereas other cells expressed them weakly (Fig 1C and 1G) or at an undetectable level (Fig 1B and 1E). On the other hand, the S100A7 protein was only expressed in a few adenocarcinoma cells. It was strongly expressed in the keratinizing cells of squamous cell carcinomas (S1 Fig). Thus, the S100A7 protein was excluded from the following analysis.

**Fig 2:**
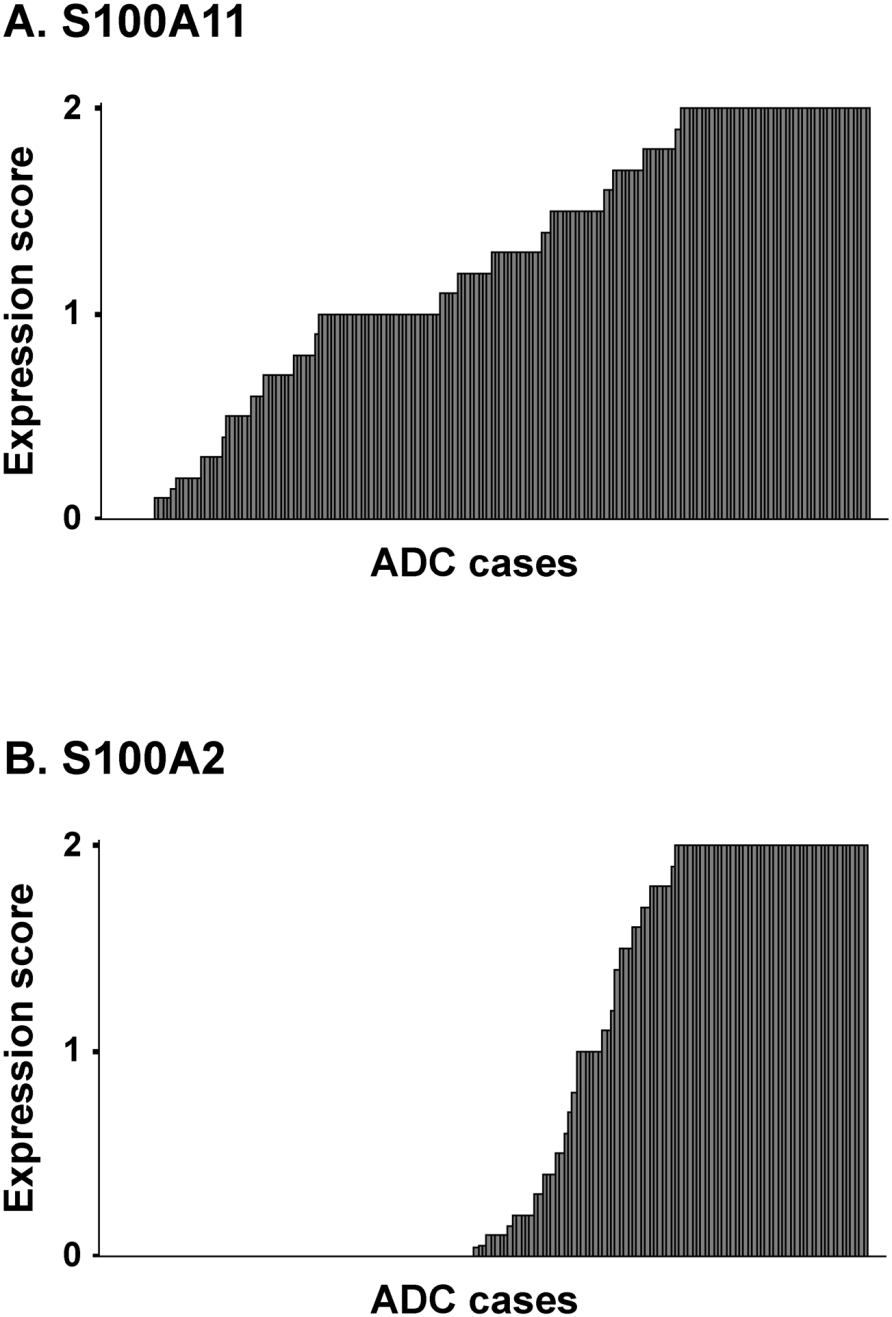
The S100A11 and S100A2 expression levels in all tumors. Median expression scores were 1.30 in S100A11 (A) and 0.10 in S100A2 (B). S100A11 was not expressed in 8 cases (A), while S100A2 was not expressed in nearly half of the cases examined (83 cases) (B). ADC, Adenocarcinoma.

The expression levels of S100A2 and S100A11 were classified into negative (level 0), weak (level 1), and strong (level 2). The weak level was defined as the level almost equivalent to that in bronchiolar epithelial cells. The strong level was defined as an unequivocally stronger level than that in bronchiolar epithelial cells. The immunohistochemical expression score was determined as the average level in a maximal tumor section (if 30%, 10%, and 60% of neoplastic cells in the maximal tumor section were negative, weak, and strong levels, respectively, the average level was calculated as “0.3x0+0.1x1+0.6x2 = 1.3”). The expression levels of S100A2 and S100A11 in all the tumors examined are shown in Fig 2A and 2B.

The validity of the immunohistochemical evaluation was supported by the Western blot analysis because the S100 protein levels evaluated by immunohistochemistry and those by Western blotting were roughly parallel (The result of a Western blot for S100A11 was shown in S2 Fig).

### S100 protein levels and clinicopathologic factors

**Fig 3:**
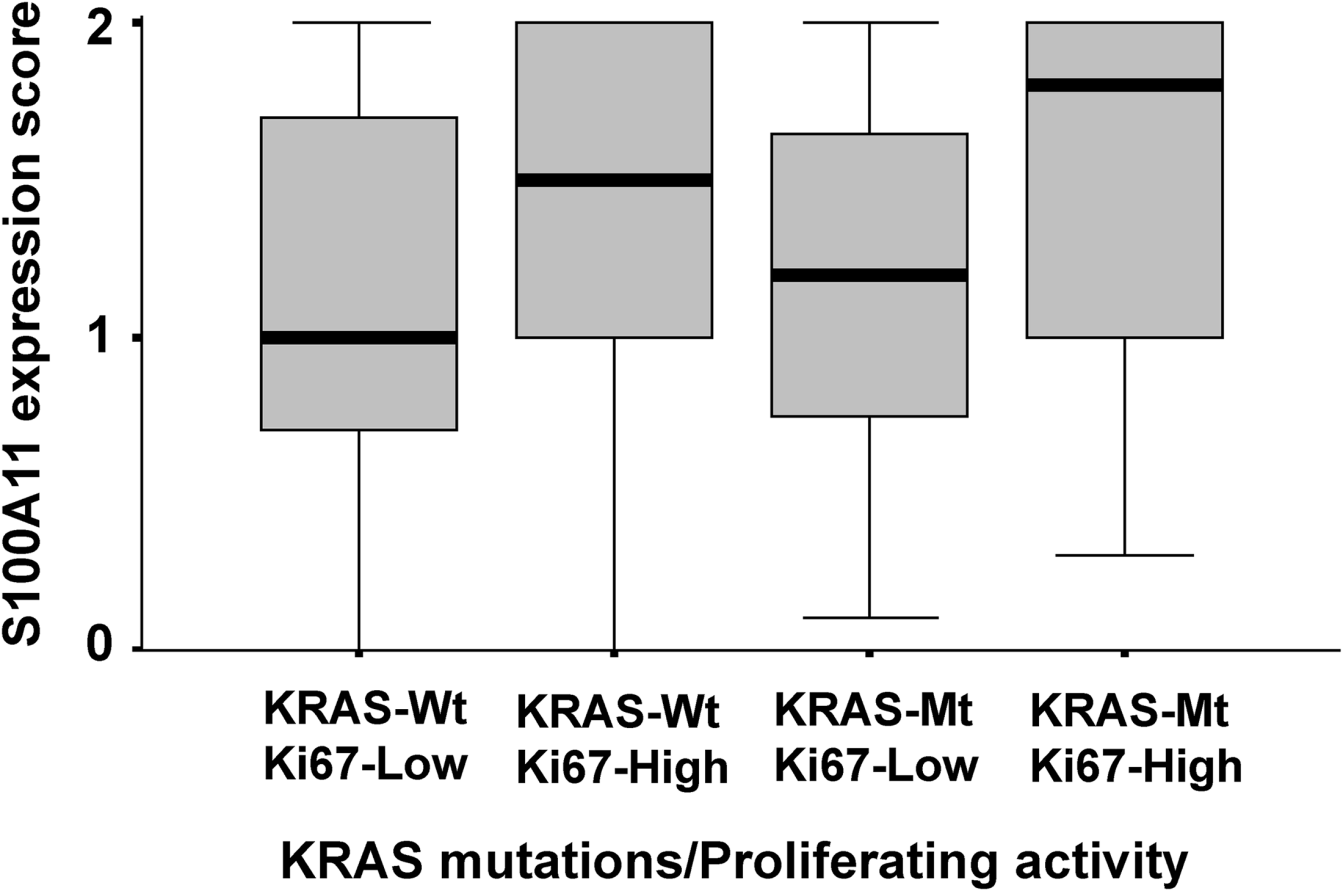
S100A11 expression levels in adenocarcinomas with KRAS mutations and proliferating activity. The thickened lines indicate the median score of S100 A11 expression, which was 1.00 in KRAS Wild-type/Ki-67 Low cases (n = 94), 1.50 in KRAS Wild-type/Ki-67 High cases (n = 62), 1.20 in KRAS Mutated-type/Ki-67 Low cases (n = 11) and 1.80 in KRAS Mutated-type/Ki-67 High cases (n = 11). S100A11 expression levels were significantly higher in adenocarcinomas with KRAS mutations and strong proliferating activity (P = 0.038 in the Kruskal-Wallis test). Wt, Wild-type; Mt, Mutated-type.

S100A11 levels were significantly higher in adenocarcinomas with KRAS mutations and strong proliferating activity (P = 0.038 in the Kruskal-Wallis test, Fig 3). They were also significantly higher in adenocarcinomas with poorly differentiated tumors (P = 0.021 in the Kruskal-Wallis test, Table 3) and lymphatic or vascular involvement (P = 0.026 in the Mann-Whitney test, Table 3). A multiple linear regression analysis showed that poorly differentiated tumors correlated with higher S100A11 levels (P = 0.039, Table 4). On the other hand, S100A2 levels were not associated with KRAS mutations, proliferating activity, or any of the other clinicopathologic factors (data not shown).

**Table 3:** Relationship between S100A11 expression and clinicopathologic characteristics of stage I lung adenocarcinomas.

Subjects	No.	Expression score (median)	P-value
**Age**			
<65	63	1.20	P = 0.617
> = 65	116	1.30	
**Gender**			
Male	89	1.20	P = 0.845
Female	90	1.30	
**Histologic Grade**			
WEL	42	1.00	P = 0.021
MOD	90	1.30	
POR	47	1.50	
**Histologic Subtype**			
AIS/MIA	42	1.00	P = 0.064
LEP	43	1.20	
CAN	59	1.50	
PAP/MPAP	17	1.40	
SOL	12	1.25	
MUC	6	1.35	
**Lymphatic or vascular involvement**			
Absent	129	1.20	P = 0.026
Present	50	1.50	

No., number of cases; WEL, well differentiated; MOD, moderately differentiated; POR, poorly differentiated carcinomas; AIS, adenocarcinoma in situ; MIA, minimally invasive adenocarcinoma; LEP, lepidic predominant; ACN, acinar predominant; PAP, papillary predominant, SOL, solid predominant; MUC, invasive mucinous adenocarcinoma.

Histologic subtypes were classified according to the 2011 IASLC/ ATS/ETS classification of lung adenocarcinoma.

P, significant level for Mann-Whitney or Kruskal-Wallis test.

**Table 4:** Multiple linear regression analysis of relationships between S100A11 expression and clinicopathologic characteristics of stage I lung adenocarcinomas.

Subjects	Coefficient B	Standard Error	Standardized Coefficient β	P-value
**Age**				
<65	1			
> = 65	2.069	9.944	0.016	0.835
**Gender**				
Male	1			
Female	2.878	9.612	0.023	0.765
**Histologic Grade**				
WEL	1			
MOD	19.767	12.240	0.156	0.108
POR	31.061	14.925	0.216	0.039
**Lymphatic or vascular involvement**				
Absent	1			
Present	13.261	11.532	0.094	0.252

WEL, well differentiated; MOD, moderately differentiated; POR, poorly differentiated carcinomas.

Histologic subtypes were excluded from factors due to the strong correlation that existed with histologic grade (Spearman’s coefficient 0.567, P<0.001).

### S100A11 protein levels and disease-free survival

**Fig 4:**
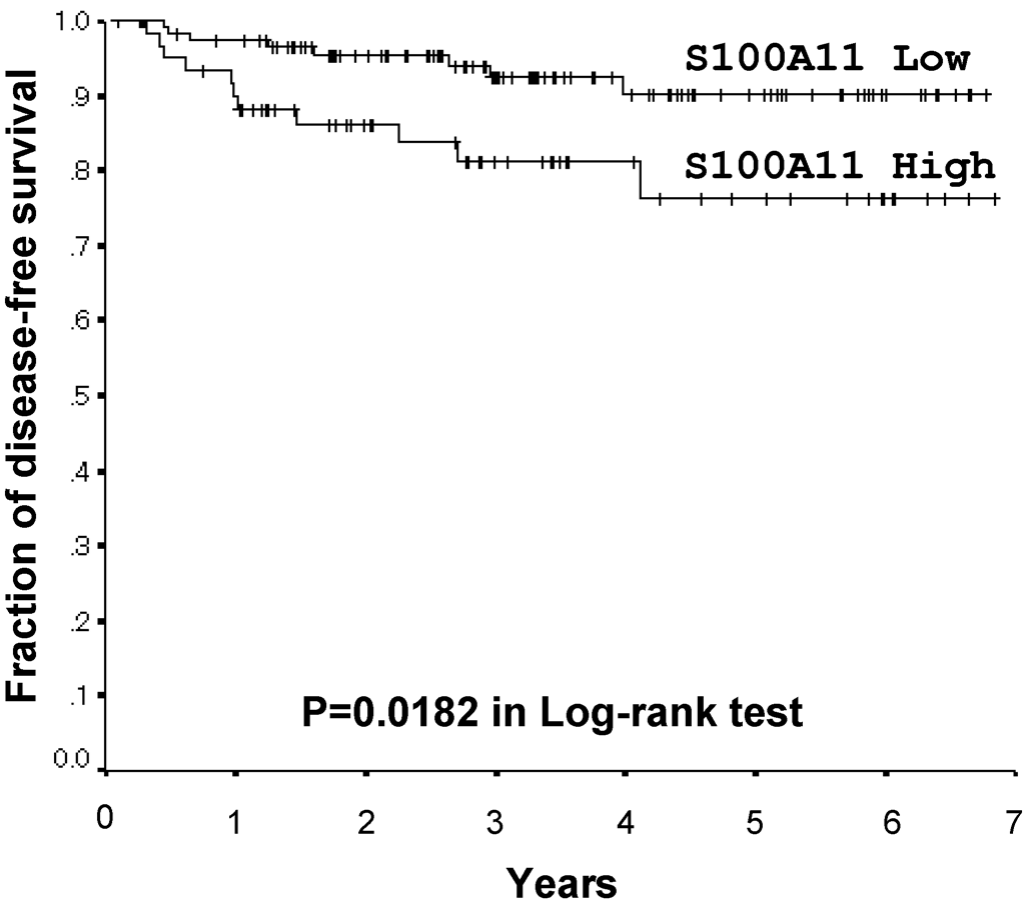
Kaplan-Meier Disease-free survival curves by S100A11 expression levels for stage I lung adenocarcinomas. Five-year disease-free survival rates were 76.3% in high-S100A11 expressers (n = 61) and 90.1% in low-S100A11 expressers (n = 118). The strong expression of S100A11 correlated with shorter disease-free survival in post-operative lung adenocarcinomas (p = 0.0182 in the Log-rank test).

Since the results obtained suggested that the up-regulation of S100A11 was involved in tumor progression, particularly in KRAS-mutated lung adenocarcinomas, its prognostic value was subsequently verified. One hundred and seventy-nine patients with lung adenocarcinomas at stage I were examined. S100A11 expression scores of <1.65 and > = 1.65 were classified as low and high based on a receiver operating characteristic curve (area under the curve 0.629, 95% confidential interval 0,501–0.757). One hundred and eighteen patients were low-expressers, while 61 were high-expressers. Five-year disease-free survival rates were 90.1% and 76.3% in low- and high-S100A11 expressers, respectively. The strong expression of S100A11 correlated with shorter disease-free survival in post-operative lung adenocarcinomas (p = 0.0182 in the Log-rank test, Fig 4). On the other hand, a correlation was not found between S100A11 expression levels and the site of recurrent disease (data not shown).

## Discussion

Among the S100 proteins (S100A2, S100A7, and S100A11) examined in the present study, S100A11 levels were significantly higher in adenocarcinomas with KRAS mutations and strong proliferating activity (Fig 3). They were also higher in adenocarcinomas with poorly differentiated tumors (Tables 3 and 4). Furthermore, the strong expression of S100A11 correlated with shorter disease-free survival (Fig 4). These results suggested that alterations in the expression of S100A11 played a role in tumor progression, particularly in KRAS-mutated lung adenocarcinomas.

S100A11, also called S100C or calgizzarin, belongs to the S100 family of multi-gene calcium-binding proteins. It is located on 1q21 with 15 other S100 family members and has a molecular weight of 11.7kDa [24]. S100 family members regulate important intracellular events such as enzyme activities, the dynamics of cytoskeletal constituents, and Ca^2+^ concentrations, in order to modulate cell growth and motility as well as differentiation [9]. The up-regulation of S100A11 has been reported in various cancers [9], such as laryngeal [10], breast [11], lung [12–13], gastric [14], colorectal [15], pancreatic [16], and prostate cancers [17], and has frequently been associated with cancer progression, implicating its oncogenic role [10–17]. Tian T et al. previously demonstrated that S100A11 was up-regulated in a highly metastatic lung cancer cell line through a proteomic analysis [13]. Wang et al. also reported that S100A11 levels increased with progression of the disease stage in colorectal cancer [15]. Our results are consistent with these findings.

However, previous studies demonstrated that S100A11 expression levels were reduced in tumors with higher malignant activity in kidney [19] and bladder cancers [20]. Memon AA et al. showed that S100A11 was down-regulated in a higher-graded cell line of bladder cancer by a proteome analysis, and the weak expression of S100A11 was associated with poor survival [20]. This discrepancy implies the complexity of cancer progression. For example, several cancer-related proteins, such as transforming growth factor-beta, have opposite functions as inhibitors or promoters in different stages of cancer progression [25]. Therefore, the potential role of the aberrant expression of S100A11 in carcinogenesis may differ among various types of malignancies.

In summary, the results of the present study suggested that the up-regulation of S100A11 played a role in tumor progression, particularly in KRAS-mutated lung adenocarcinomas. The biological function of S100A11 still remains unclear. Further investigations are warranted in order to elucidate the mechanisms by which S100A11 promotes the progression of lung cancer.

## Supporting Information

S1 Fig Immunohistochemical examination of S100A7 protein expression levels in tumors and non-tumorous epithelia from lung cancer patients undergoing surgical resection. Representative photographs from normal bronchioles (A), adenocarcinoma (B) and squamous cell carcinoma (C) are shown. S100A7 was not expressed in the normal epithelial cells of bronchioles (A), and was only expressed in a few adenocarcinoma (ADC) cells (B). On the other hand, it was strongly expressed in the keratinizing cells of squamous cell carcinomas (SQC) (C).
(TIFF)

S2 Fig Protein lysates from tumors (T) and non-tumorous tissues (N) of lung adenocarcinoma tissues of patients undergoing surgical resection were subjected to a Western blot analysis for S100A11 and β-actin (ACTB) in the representative cases (A). The signal intensities of the bands were evaluated by NIH imaging. S100A11 levels were normalized to those of ACTB. Normalized levels are shown (B). The immunohistochemical expression of S100A1 in the same tumors was shown (C). S100 protein levels evaluated by immunohistochemistry and those by Western blot were roughly parallel. Cs, Case.
(TIFF)
