# Supplementary figures and images for: Up-Regulation of S100A11 in Lung Adenocarcinoma – Its Potential Relationship with Cancer Progression
Source: PLoS One. 2015 Nov 6;10(11):e0142642. doi: 10.1371/journal.pone.0142642 (PMC4636248; doi:10.1371/journal.pone.0142642)

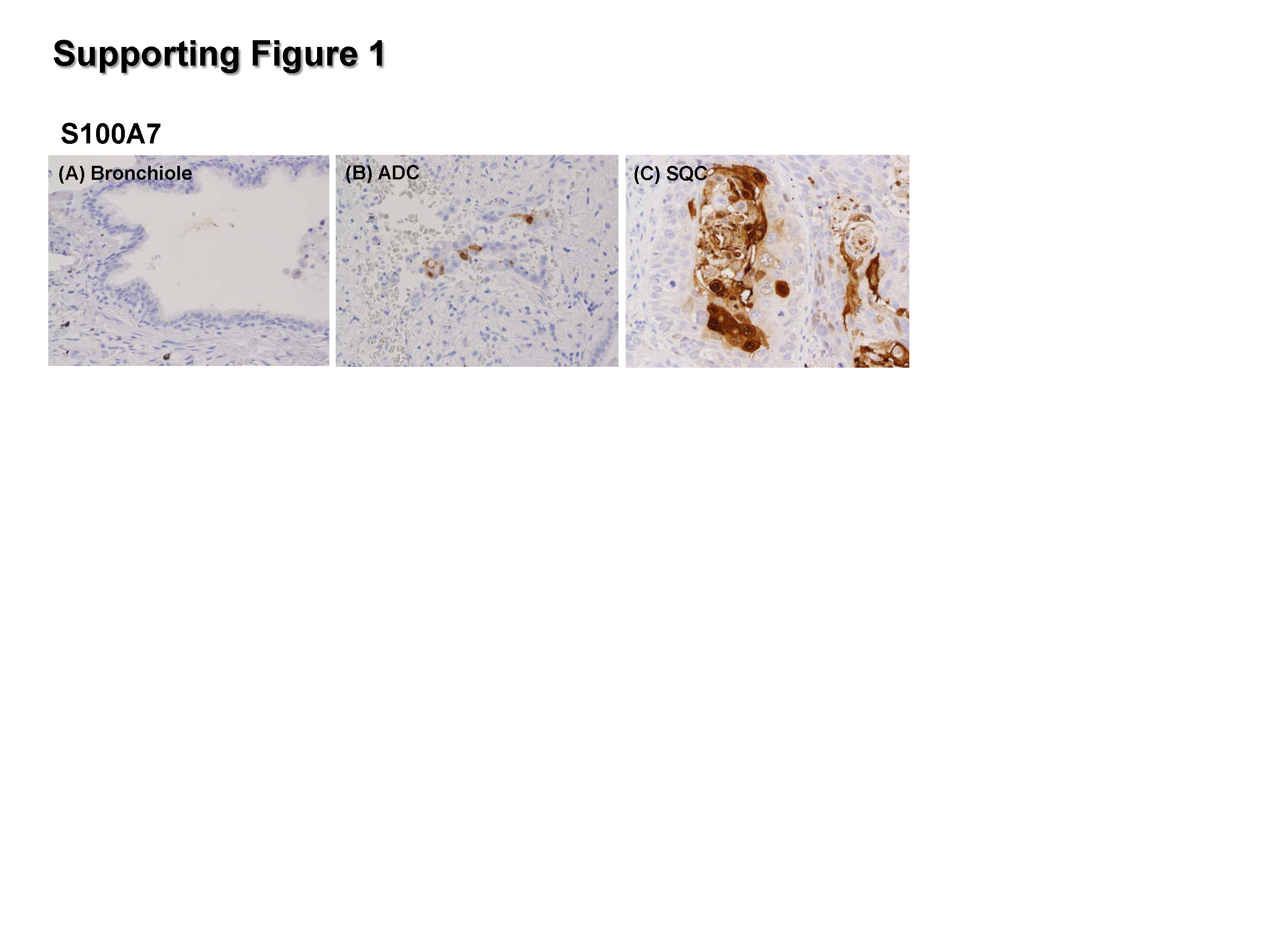

Supplement: S1 Fig — Representative photographs from normal bronchioles (A), adenocarcinoma (B) and squamous cell carcinoma (C) are shown. S100A7 was not expressed in the normal epithelial cells of bronchioles (A), and was only expressed in a few adenocarcinoma (ADC) cells (B). On the other hand, it was strongly expressed in the keratinizing cells of squamous cell carcinomas (SQC) (C). (TIFF) [file pone.0142642.s001.tiff]

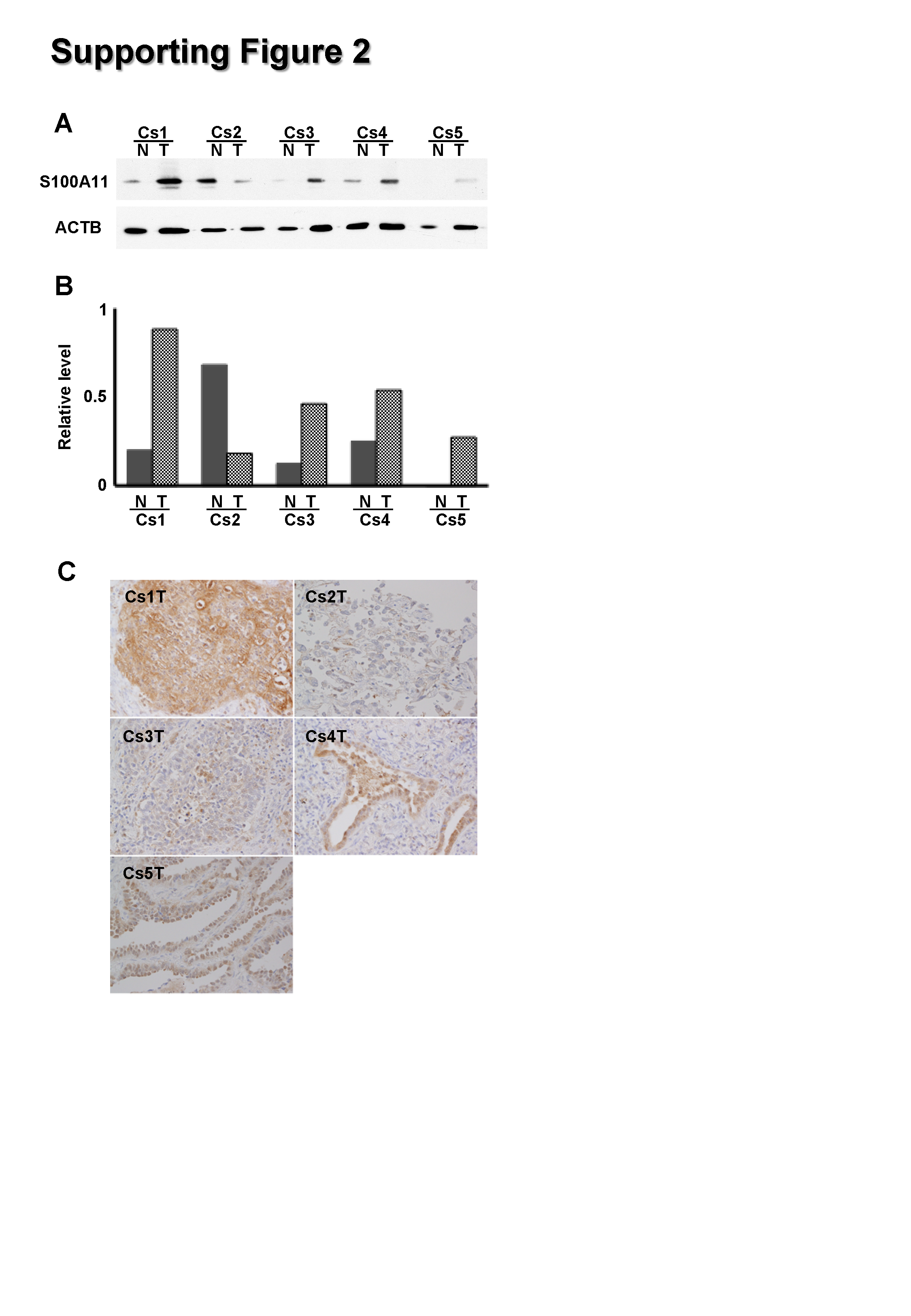

Supplement: S2 Fig — Protein lysates from tumors (T) and non-tumorous tissues (N) of lung adenocarcinoma tissues of patients undergoing surgical resection were subjected to a Western blot analysis for S100A11 and β-actin (ACTB) in the representative cases (A). The signal intensities of the bands were evaluated by NIH imaging. S100A11 levels were normalized to those of ACTB. Normalized levels are shown (B). The immunohistochemical expression of S100A1 in the same tumors was shown (C). S100 protein levels evaluated by immunohistochemistry and those by Western blot were roughly parallel. Cs, Case. (TIFF) [file pone.0142642.s002.tiff]
